# Mycogenic Selenium Nanoparticles as Potential New Generation Broad Spectrum Antifungal Molecules †

**DOI:** 10.3390/biom9090419

**Published:** 2019-08-28

**Authors:** Shreya M. Joshi, Savitha De Britto, Sudisha Jogaiah, Shin-ichi Ito

**Affiliations:** 1 Laboratory of Plant Healthcare and Diagnostics, PG Department of Studies in Biotechnology and Microbiology, Karnatak University, Pavate Nagar, Dharwad 580 003, Karnataka, India; 2 Division of Biological Sciences, School of Science and Technology, The University of Goroka, Goroka 441, Papua New Guinea; 3 Laboratory of Molecular Plant Pathology, Department of Biological and Environmental Sciences, Graduate School of Sciences and Technology for Innovation, Yamaguchi University, Yamaguchi 753-8515, Japan; 4 Research Center for Thermotolerant Microbial Resources (RCTMR), Yamaguchi University, Yamaguchi 753-8515, Japan

**Keywords:** *Trichoderma atroviride*, selenium nanoparticles, broad spectrum, antifungal agents

## Abstract

The current challenges of sustainable agricultural development augmented by global climate change have led to the exploration of new technologies like nanotechnology, which has potential in providing novel and improved solutions. Nanotools in the form of nanofertilizers and nanopesticides possess smart delivery mechanisms and controlled release capacity for active ingredients, thus minimizing excess run-off to water bodies. This study aimed to establish the broad spectrum antifungal activity of mycogenic selenium nanoparticles (SeNPs) synthesized from *Trichoderma atroviride,* and characterize the bioactive nanoparticles using UV–Vis spectroscopy, dynamic light scattering (DLS), Fourier transform infrared (FT-IR), X-ray diffraction (XRD), scanning electron microscopy-energy dispersive X-ray spectroscopy (SEM-EDS), and high-resolution transmission electron microscopy (HR-TEM). The synthesized nanoparticles displayed excellent in vitro antifungal activity against *Pyricularia grisea* and inhibited the infection of *Colletotrichum capsici* and *Alternaria solani* on chili and tomato leaves at concentrations of 50 and 100 ppm, respectively. The SEM-EDS analysis of the bioactive SeNPs revealed a spherical shape with sizes ranging from 60.48 nm to 123.16 nm. The nanoparticles also possessed the unique property of aggregating and binding to the zoospores of *P. infestans* at a concentration of 100 ppm, which was visualized using light microscope, atomic force microscopy, and electron microscopy. Thus, the present study highlights the practical application of SeNPs to manage plant diseases in an ecofriendly manner, due to their mycogenic synthesis and broad spectrum antifungal activity against different phytopathogens.

## 1. Introduction

Nanoparticles are gaining rapid momentum in plant disease management due to their large surface area to volume ratio, which allows them to establish better contact with microorganisms, thus leading to enhanced antimicrobial activity [1,2]. The synthesis of nanoparticles by fungi, also called myconanotechnology, is an emerging branch of nanotechnology that has the potential to meet the crucial needs of disease control and crop management [2,3,4]. The application of myco-nanostructures can effectively reduce the excessive use of agrochemicals and site targeted active ingredients [4,5,6]. The bio-reduction of metal oxides to their elemental form is catalyzed mainly by the extracellular enzymes and metabolites released by the fungal organisms that form the basis of the myco-synthesis of nanoparticles in an eco-friendly manner [7]. During this process, the toxicity of the metal ions is negated to the non- toxic metallic nanoparticles [8]. For instance, selenium is found to be less toxic and more biologically active [9,10,11] in its reduced nano-form when compared to its other chemical forms such as sodium selenite and selenium sulfide [12]. In their amorphous forms, selenium nanoparticles (SeNPs) possess peculiar photoelectric, semiconducting, and X-ray-sensing properties [13]. Additionally, SeNPs are being explored explicitly for their anti-microbial [11,14,15] antioxidant, anti-cancerous, and anti-inflammatory properties [11,16]. In addition, SeNPs are widely used in nutritional supplements, medical apparatus, and nanotherapeutics [11]. In biological systems, this micronutrient metalloid is reported to be the main component of selenoenzymes such as glutathione peroxidase, iodothyronine deiodinase, and thioredoxin reductase, which are involved in antioxidant defense, detoxification, and metabolism, respectively [17,18]. The biological activities and good adsorptive ability of SeNPs can be attributed to the interactions between the nanoparticles and functional groups present in proteins such as NH, C=O, COO–, and C–N [9].

SeNPs derived from *Trichoderma* have recently been demonstrated to control pearl millet downy mildew disease and improve plant growth under greenhouse conditions [19]. The mycosynthesized nanoparticles could inhibit the sporulation of pathogens on infected host leaves. Interestingly, it has also been observed that the disease protection ability of *Trichoderma* increased dramatically in the presence of SeNPs. Thus, from these studies, it can be postulated that SeNPs can be a potential tool in integrated plant disease management. *Trichoderma* is a free living, asexually reproducing, root colonizing fungus that is known to possess mycoparasitic and antimycotic activities against fungal pathogens [20,21]. Moreover, the biocontrol agent triggers systemic and localized resistance in plants against a large number of biotic stresses [21,22]. This induction of disease resistance is mainly attributed to the presence of ABC transporters that are involved in the secretion of antibiotics and cell wall degrading enzymes [23]. The emergence of late blight of tomato in south-western regions of India was reported by Chowdappa et al. [24], the disease being caused by the oomycete fungi *Phytophthora infestans,* which causes blight on the leaf, fruit, and stem, leading to rotting of the whole plant. The disease has the potential to cause up to 100% yield loss. As this fungus is heterothallic and reproduces sexually, the pathogen evolves rapidly, thus limiting the effectiveness of conventional fungicides for the control of late blight disease.

In this study, the aim was to synthesize SeNPs from *Trichoderma* and establish the broad spectrum activity of the myconanoparticles against different fungal plant pathogens. Initially, three different fractions of *Trichoderma* broth culture were subjected to nanoparticle synthesis by the reduction of sodium selenite to elemental selenium. The broad spectrum activity of the biosynthesized nanoparticles was demonstrated by in vitro antifungal activity against *Pyricularia grisea* and *in vitro* leaflet assay against *Colletotrichum capsici* and *Alternaria solani.* The bioactive nanoparticles were further characterized by UV–Vis absorption spectroscopy, zeta potential or DLS, Fourier transform infrared (FT-IR), X-ray diffraction (XRD), scanning electron microscopy-energy dispersive X-ray spectroscopy (SEM-EDS) and high resolution transmission electron microscopy (HR-TEM) and high resolution transmission electron microscopy (HR-TEM). The potential role of nanoparticles in the prevention of the sporulation of late blight of tomato was also carried out by treating the *P. infestans* zoospores with different concentrations of SeNPs, followed by observations under light microscopy, atomic force microscope, and SEM.

## 2. Materials and Methods

### 2.1. Collection and Maintenance of Trichoderma and Pathogenic Organisms

The *Trichoderma atroviride* (Tri_AtJSB2) sp. used for the mycosynthesis of SeNPs was previously identified by Jogaiah et al. [20] and was maintained on potato dextrose agar (PDA) at 23 ± 2 °C. The *P. infestans* isolate was obtained from the Indian Institute of Horticultural Research, Bengaluru, India and maintained on rye agar A at 18 °C and 98% relative humidity. Of the other phytopathogens, *P. grisea* was isolated from pearl millet (*Pennisetum glaucum* (L.) R. Br.), cultivar GHB558 at the village Kendoor, Pathogen; *A. solani* was isolated from tomato (*Solanum lycopersicum* Mill.), cultivar Durga near Hebsur farm; while the isolate of *C. capsici* was gifted by the University of Agriculture Sciences, Dharwad.

### 2.2. Preparation of Fungal Extracts

The mycosynthesis of selenium nanoparticles was performed using three different fractions of the fungal culture as described previously by Nandini et al. [19]. For the synthesis of SeNPs from *Trichoderma*, the fungi was grown in 500 mL of potato dextrose broth (PDB) under dark and static conditions for a period of seven days at 23 ± 2 °C. The intact fungal mat that formed was separated from the culture filtrate (CF) by filtration using vacuum pump (Buchi, V-700, Switzerland). The obtained mat was washed repeatedly with 100 mL of sterile distilled water. Five grams of wet biomass of mat were crushed to a fine powder with liquid nitrogen and suspended in about 30 mL of sterile distilled water. The suspension was subjected to sonication at 20 micron amplitude for 60 s with periodic intervals of 30 s (Sonics Vibra Cell, Melville, New York USA). The sonicated solution was centrifuged at 10,000 rpm at 4 °C for 10 min and cell lysate (supernatant) was separated from the cell wall debris (pellet).

### 2.3. Biosynthesis of Selenium Nanoparticles (SeNPs)

The selenium nanoparticles were synthesized using the three fractions namely, culture filtrate (CF), cell lysate (CL), and cell wall debris (CW) of *T. atroviride*. One hundred milliliters of 25 mM sodium selenite solution prepared in sterile distilled water was added to 25 mL each of culture filtrate and cell lysate and 5 g of cell wall debris. The synthesis was carried out at 23 ± 2 °C for seven days and with constant rotation at 200 rpm. The reaction mixture was observed for any color change at regular intervals. The nanoparticles that formed were collected at different time intervals by centrifuging the collected solution at 10,000 rpm for 10 min, and the precipitates obtained were washed with sterile distilled water. Cell free extract (without sodium selenite) was maintained as a control under the same conditions [19].

### 2.4. Antifungal Activity of SeNPs

The antifungal activities of SeNPs were determined by the measurement of the radial growth of pathogens on PDA plates amended with different concentrations of the synthesized nanoparticles. Briefly, the synthesized nanoparticles were dissolved in sterile distilled water in appropriate amounts to obtain a final concentration of 50, 100, and 200 ppm. The solutions were added to flasks containing sterilized PDA media and poured into petri plates. Each plate was seeded with 5 mm mycelial plug of *P. grisea* that was taken from the edge of the seven days old culture. Plates without nanoparticles served as the control. The petri plates were incubated at 22 ± 2 °C for seven days and the mycelial growth was measured at regular intervals. Growth inhibition was expressed as the percentage of inhibition of radial growth relative to the respective control [25]. 

### 2.5. In Vitro Leaflet Assay 

Antifungal activities of SeNPs were also demonstrated using one-month-old chili (*Capsicum annuum* L.) cultivar ArkaKhyati and tomato leaves to establish the inhibitory activity of nanoparticles based on the spread or severity of disease symptoms. The plants were maintained under greenhouse conditions at 20 to 32 °C and 60 to 70% RH. The detached healthy leaves were surface sterilized with 2% sodium hypochlorite solution and moistened with different concentrations of SeNPs (10, 25, 50, and 100 ppm). After drying the leaves, 5 mm each of agar plugs containing plant pathogen *C. capsici*, which causes anthracnose disease in chili, and *A. solani*, which causes early blight of tomato, were placed on healthy chili and tomato leaves, respectively. The treated and inoculated leaves were maintained in a moist chamber for seven days at 22 ± 2 °C under dark conditions. The inhibition of disease was evaluated on the basis of the diameter of the infected region [4]. 

### 2.6. Bioactivity Guided Characterization of SeNPs

The formation of the SeNPs was initially confirmed by a color change from pale yellow to brick red after 24 h of incubation. Next, a UV–Vis spectroscopic analysis was performed over wavelengths from 200 to 400 nm and a resolution of 1 nm at room temperature. The zeta potential and particle distributions of the SeNPs were determined by dynamic light scattering (DLS). The probable interactions between the formed nanoparticles and the fungal metabolites were identified using FT-IR absorption spectrum (Perkin Elmer spectrometer). For this process, a small quantity of nanoparticles was crushed with potassium bromide to form the pellet. The analysis was carried out in the range of 500–4000 nm. The crystalline structure of the nanoparticle was demonstrated by studying the X-ray diffraction patterns using a Bruker Model D8 Advance. A small quantity of each nanoparticle was smeared on a low background sample holder (amorphous silica holder) and fixed on the sample stage in the goniometer. The voltage and current was set to 40 mV, 35 mA, respectively and the goniometer was set to B–B geometry. The intensities of the diffracted rays were then recorded in the range of 10–80 °C. Furthermore, the surface morphology and the dimensions of nanoparticles were studied by scanning electron microscopy-energy dispersive X-ray spectroscopy (SEM-EDS) and high-resolution transmission electron microscopy (HR-TEM). The SEM-EDS was carried by smearing the sample on a small piece of adhesive carbon tape that was fixed on a brass stub. The sample was then subjected to gold coating using a sputtering unit (model: JFC1600) for 10 s at 10 mA of current. The gold coated sample placed in the SEM chamber (Jeol, JSM 6390LA) and secondary electron/back scattered electron images were recorded. Elemental analysis was carried out in the scanned area/point/line using an EDAX detector (OXFORD XMX N). Finally, HR-TEM was performed by suspending an extremely small amount of material in water/ethanol (just enough to obtain a slightly turbid solution). The solution was homogenized using an ultrasonicator to obtain a uniform distribution of the particles. A drop of the solution was pipetted out and casted on carbon-coated grids. The grid was dried and fixed in the specimen holder for observation (Jeol Model JM 2100).

### 2.7. Inhibitory Action of Selenium Nanoparticles Against P. infestans

The zoospores of *P. infestans* were harvested from 12-day-old cultures grown on rye agar A media by flooding the petri plates with 15 mL of sterile distilled water. The final concentration was adjusted to 1.4 × 10^7^ zoospores/mL using a hemocytometer. A total of 100 μL of this zoospore suspension was treated with 100 ppm of the SeNPs for 15 min and kept under dark conditions at room temperature. Copper oxychloride (100 ppm) and sterile distilled water were also applied to 100 μL zoospore suspension under the same conditions as positive and negative controls, respectively [26]. The probable effect of the nanoparticles on the zoospores were first observed under a light microscope and further investigated by atomic force microscopy and scanning electron microscopy.

### 2.8. Statistical Analysis

Data obtained from three replicates were analyzed for each experiment and subjected to analysis of variance (ANOVA) using SPSS Inc. Chicago, IL, USA 18.0. The significant effects of each treatment were determined by F values (*P* < 0.001) and the means from each treatments were separated by Tukey’s honestly significant differences (HSD) test.

## 3. Results

### 3.1. Biosynthesis of Selenium Nanoparticles

**Figure 1:**
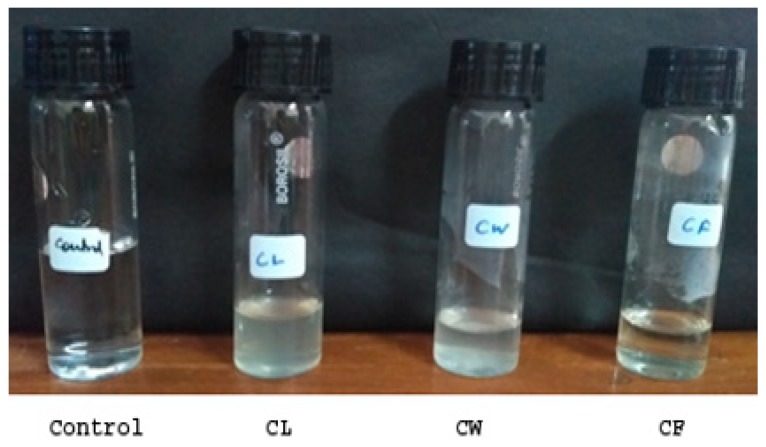
Synthesis of SeNPs from different fractions of *Trichoderma atroviride* culture lysate (CL), cell wall debris (CW), and culture filtrate (CF) at time zero.

**Figure 2:**
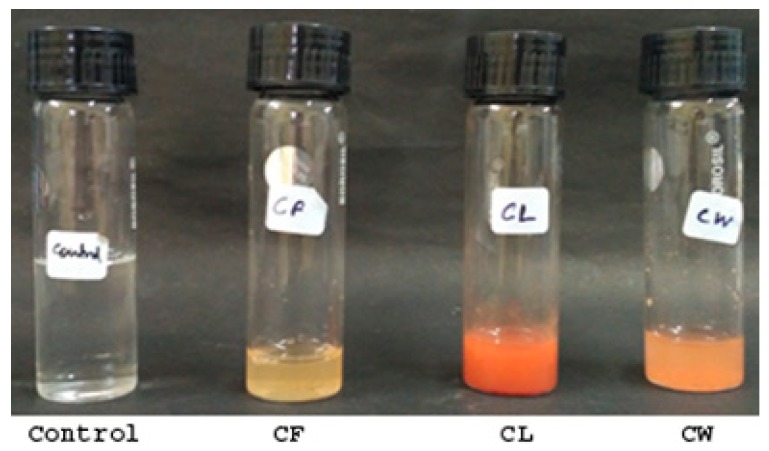
Mycosynthesized SeNPs from different fractions of *T. atroviride* culture filtrate (CF), culture lysate (CL), and cell wall debris (CW) exhibiting color change (colorless to dark brick organe) after a 24 h time interval.

The mycosynthesis of SeNP was carried out by the reduction of sodium selenite (+IV oxidation state) to elemental selenium by three different fractions of *T. atroviride* broth culture. All three fractions, namely, CF, CL, and CW, when added to a 25 mM sodium selenite solution, and produced a similar characteristic brick orange color after 24 h of incubation at 22 ± 2 °C. The final weight of the harvested nanoparticles derived from the culture filtrate was found to be 37.6 mg. Thus, both intracellular and extracellular synthesized nanoparticles were considered for initial characterization studies. For all of the biological studies, nanoparticles derived from the culture filtrate (CF) were utilized (Figure 1 and Figure 2).

### 3.2. Antifungal Activity

**Figure 3:**
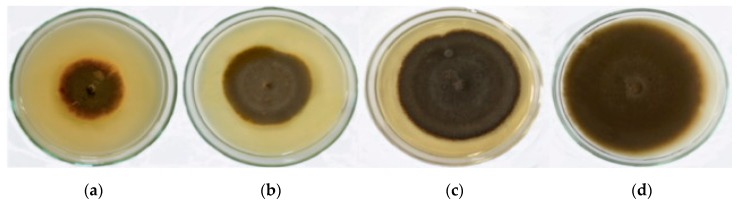
In vitro antifungal activity of mycogenic SeNPs against *Pyricularia grisea* on PDA plates at different concentrations: (**a**) 200 ppm; (**b**) 100 ppm; (**c**) 50 ppm; (**d**) control.

The antifungal activity of the nanoparticles was determined against *Pyricularia grisea*, which causes blast disease in pearl millet. The incubation was carried out for seven days, on the fifth day at 23 ± 2 °C, it was observed that very low concentrations of SeNPs at 100 and 200 ppm could successfully inhibit the fungal growth up to 52.4 and 36.8 mm, respectively (Figure 3 and Table 1).

### 3.3. In Vitro Leaflet Assay

**Figure 4:**
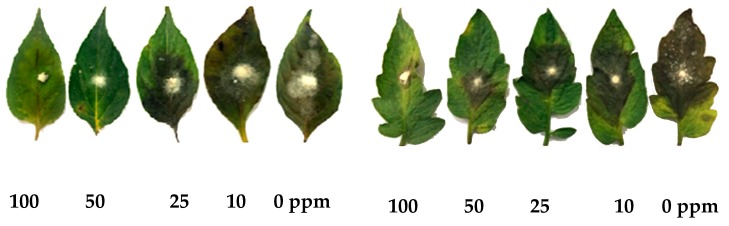
*In vitro* antifungal leaflet assay of one-month-old chili and tomato leaves primed with various concentrations of mycogenic SeNPs (100, 50, 25, and 10 and 0 ppm), and then artificially inoculated with *Collectotrichum capsici* and *Alternaria solani*.

The leaflet assay was performed using one-month-old healthy chili and tomato leaves that were treated with different concentrations of SeNPs (10, 25, 50, and 100 ppm), dried at room temperature and inoculated with *C. capsici* and *A. solani*. It was observed that high concentrations of SeNPs could effectively inhibit the spread of the infection under in vitro conditions. The fungal growth expansion was inhibited to the maximum extent at concentrations of 50 and 100 ppm, respectively, in comparison with the control (0 ppm) where the growth of the fungal infection and necrotic lesions clearly spread on the inoculated leaves. Thus, SeNPs could efficiently suppress the spread of fungal infection on chili and tomato leaves (Figure 4).

### 3.4. Bioactivity Guided Characterization of SeNPs

**Figure 5:**
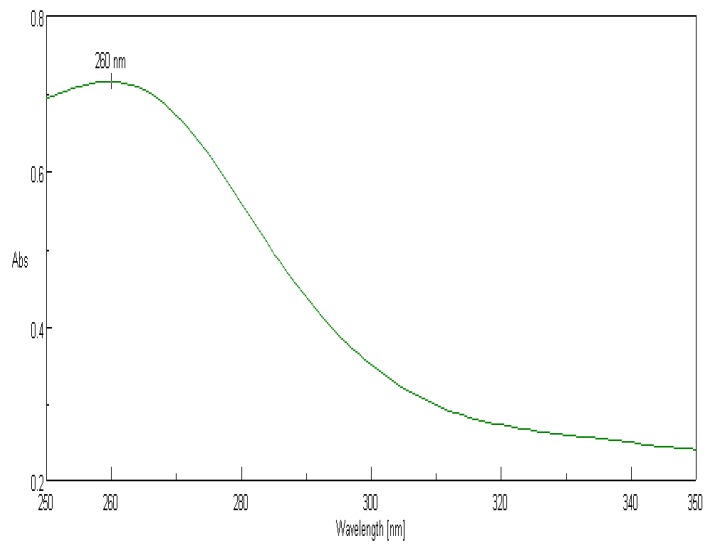
UV–Vis absorbance of mycogenic SeNPs synthesized by the culture filtrate fraction of *Trichoderma atroviride* showing a peak at 260 nm.

The bioactive SeNPs were further characterized for their size, shape, charge, and functional groups associated with them. The formation of selenium nanoparticles was initially detected by a change in color from pale yellow to a dark brick orange, caused by the reduction of sodium selenite to elemental selenium by the reducing agents present in different fractions of *Trichoderma*. A two milliliter SeNP suspension was subjected to UV–Vis absorbance spectra in the range of 200–400 nm and a peak was obtained at 260 nm (Figure 5), indicating the synthesis of SeNPs.

**Figure 6:**
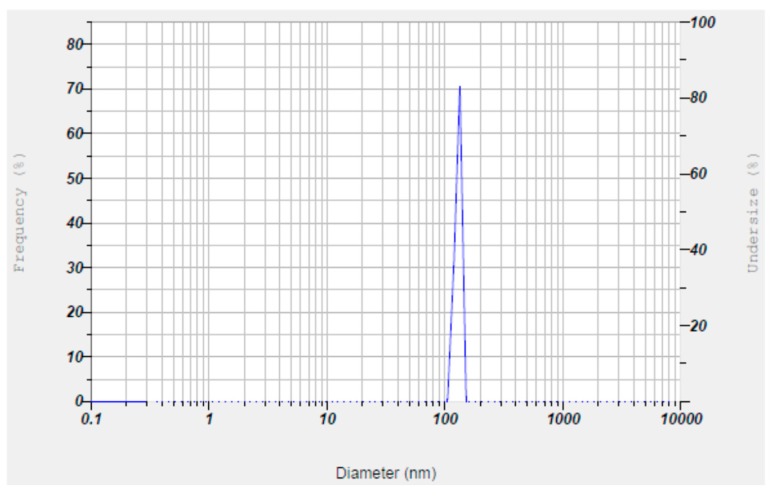
Z-average size of synthesized mycogenic SeNPs as measured by the dynamic light scattering method.

**Figure 7:**
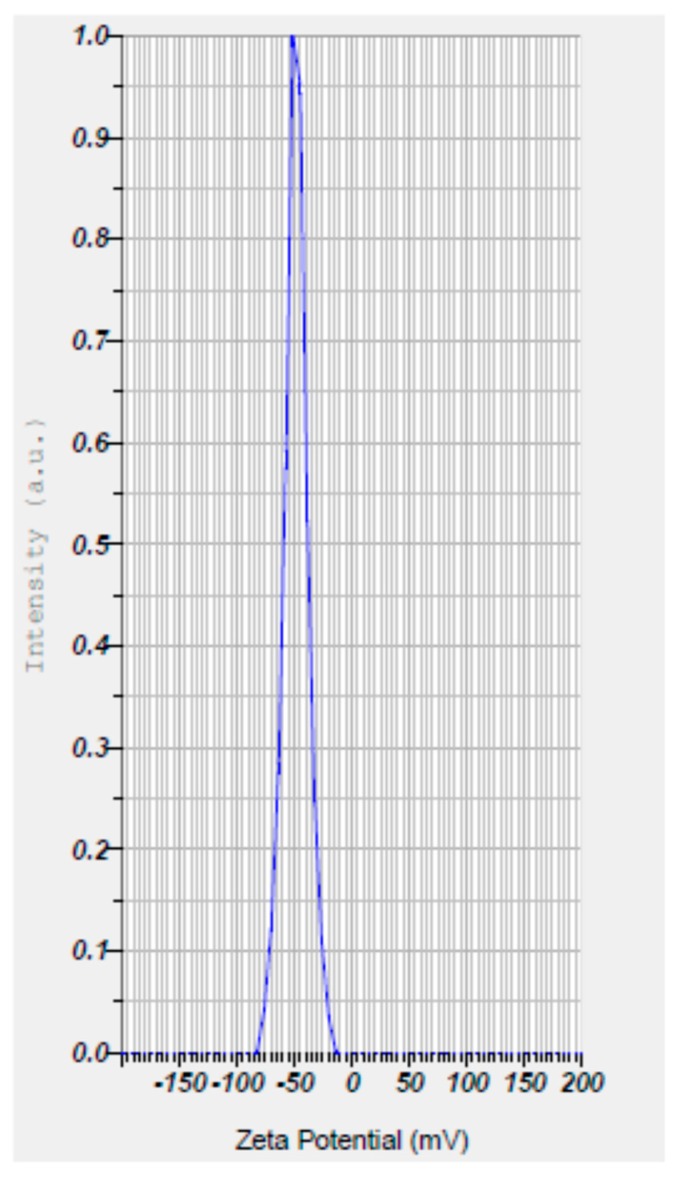
Zeta potential of mycogenic SeNPs showing a high negative potential of −40.1 mV.

**Figure 8:**
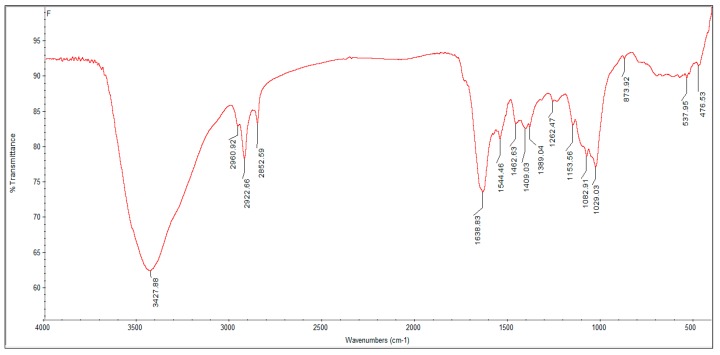
Fourier transform infrared (FT-IR) spectrum of SeNPs showing different functional groups involved in the reduction process with % transmittance.

The Z-average size and zeta potential of the nanoparticles were also studied. The Z-average size of the nanoparticles was found to be in the range of 93.2 nm to 98.5 nm, with zeta potential values ranging from −49.3 mV to −43.7 mV (Figure 6 and Figure 7; Table 2), thus indicating a uniform size and stability due to increased repulsions between the particles due to their high negative charges. The FT-IR spectrum of SeNP revealed two major peaks at 3427.88 cm^−1^ and 1638.83 cm^−1^, which corresponded to the –OH and –NH stretches (Figure 8), indicating the presence of carboxylic and amide groups, respectively. Thus, the presence of these functional groups render the SeNPs’ stability and also serve as reducing agents in the conversion of sodium selenite to elemental selenium.

**Figure 9:**
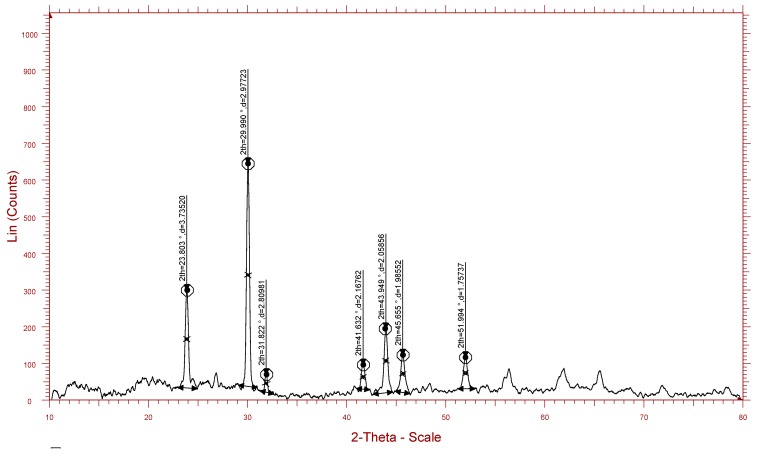
X-ray diffraction pattern of mycogenic SeNPs. Major peaks were obtained at 2θ values of 23.803, 29.990, 31.822, 41.632, 43.949, 45.655, and 51.994, corresponding to (1, 0, 0), (1, 1, 0), and (1, 1, 1) sets of lattice plane.

**Figure 10:**
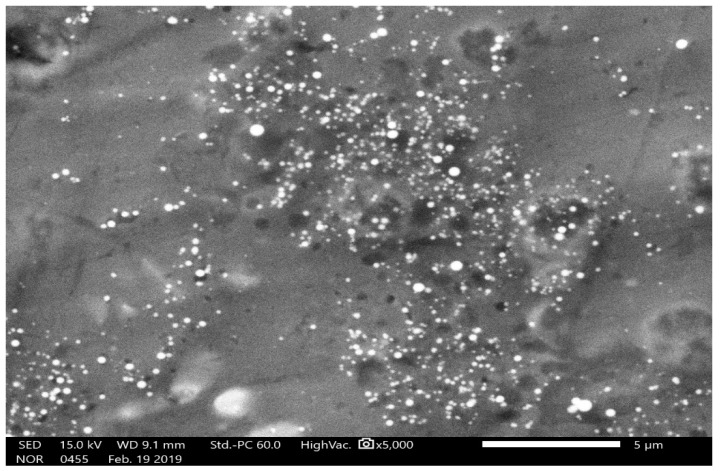
Scanning electron microscopic image mycogenic SeNPs depicting their spherical shape. The white bar indicates 5 μm.

**Figure 11:**
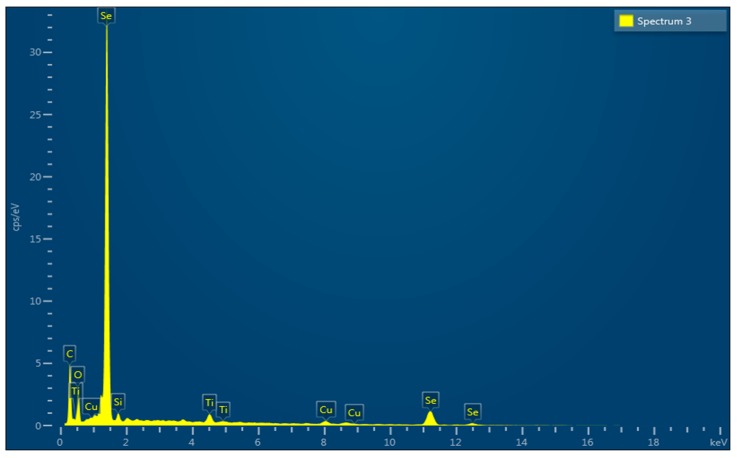
Energy dispersive X-ray spectroscopy of SeNPs depicting selenium as the major element.

**Figure 12:**
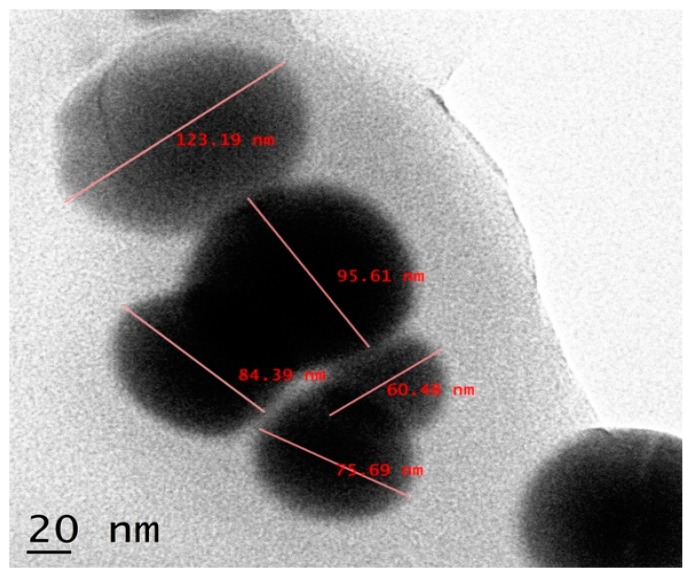
High-resolution transmission electron microscopy (HR-TEM) of SeNPs derived from the culture filtrate of *T. atroviride* depicting spherical nanoparticles ranging from 60.45 nm to 123.19 nm. Bar scale: 20 nm.

The crystalline structure of the nanoparticles was examined by X-ray diffraction. The Bragg reflections with 2θ values of 23.803, 29.990, 31.822, 41.632, 43.949, 45.655, and 51.994 were observed (Figure 9). These values corresponded to (1, 0, 0), (1, 1, 0), and (1, 1, 1) sets of lattice planes, thus depicting the crystalline nature of biosynthesized SeNPs. The surface morphology, elemental composition, size, and dimensions of the bioactive nanoparticles were investigated by SEM-EDS and HR-TEM. The nanoparticles were found to be spherical in shape (Figure 10), and selenium consisted of one of the major elements (Figure 11). The size of the nanoparticles measured by HR-TEM ranged from 60.48 nm to 123.16 nm (Figure 12).

### 3.5. Inhibitory Action of Selenium Nanoparticles Against P. infestans

**Figure 13:**
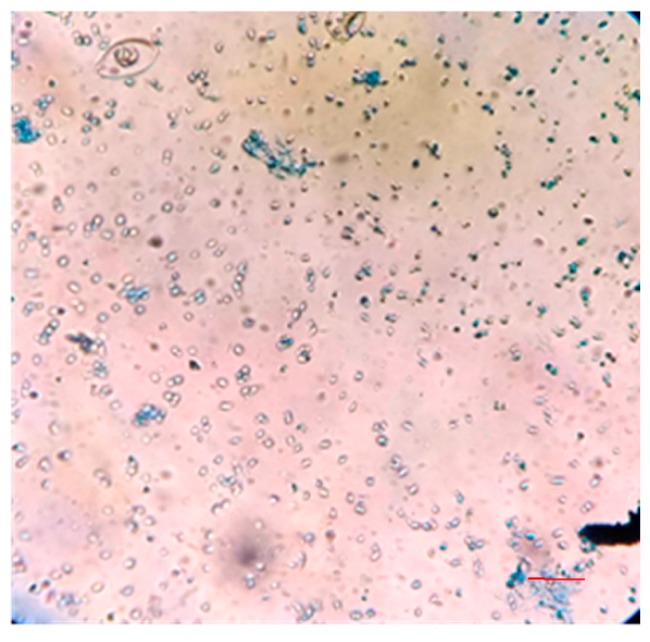
Zoospores of *P. infestans* without nanoparticle treatment as observed under 10× magnification of light microscope. The red bar scale indicates 5 µm.

**Figure 14:**
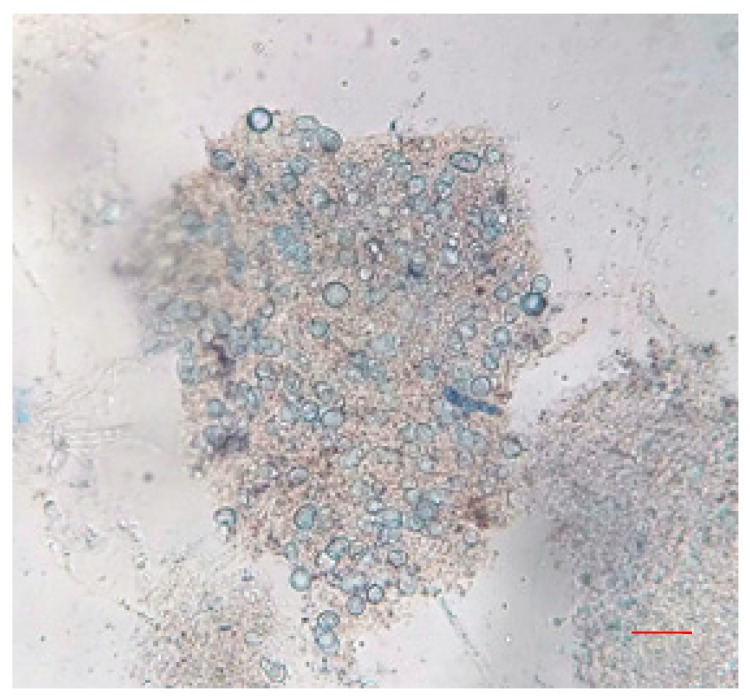
Zoospores of *P. infestans* showing extensive aggregation upon treatment with 100 ppm of SeNPs as observed under 40× magnification under a light microscope. The red bar scale indicates 5 µm.

**Figure 15:**
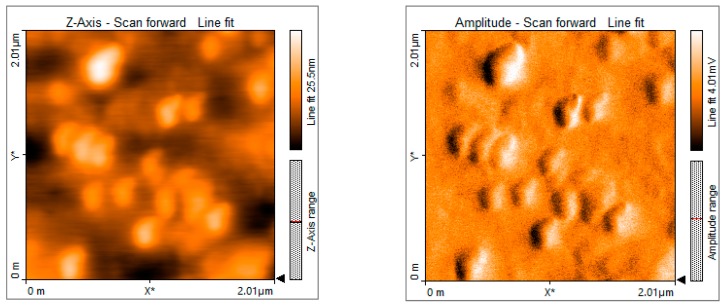
Atomic force microscopic images showing *P. infestans* zoospores without nanoparticle treatment.

**Figure 16:**
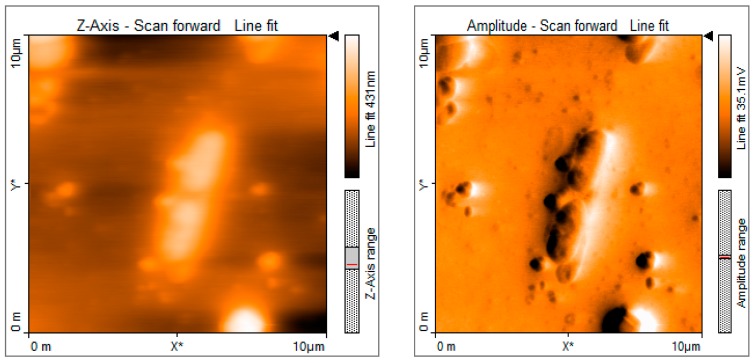
Atomic force microscopic images showing the binding of nanoparticles on the surface of *P. infestans* zoospores when treated with 100 ppm concentration of mycosynthesized SeNPs.

**Figure 17:**
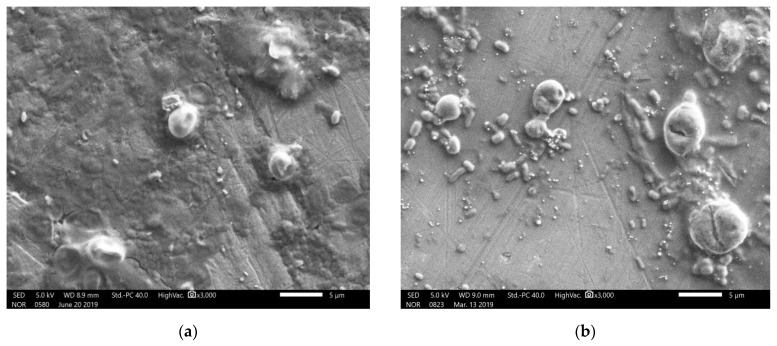
Scanning electron micrographs of *P. infestans* zoospores. (**a**) Untreated and (**b**) Treated with SeNPs at 100 ppm concentration. The white bars indicate 5 μm.

After the treatment of the 100 μL zoospore suspension with nanoparticles and fungicide, the treated suspensions were incubated in the dark for 15 min at room temperature. A drop of this suspension was placed on a cavity slide and observed by light microscope under 10× and 40× magnification. Compared to the controls (Figure 13), the nanoparticle treated (100 ppm concentration) zoospores exhibited a high aggregation of spores (Figure 14). Furthermore, this binding phenomenon was ascertained by atomic force microscopy (Figure 15 and Figure 16) and SEM (Figure 17). Thus, from these observations, it can be concluded that the mycosynthesized selenium nanoparticles have the potential to inhibit the spread of zoospores and thus reduce the occurrence of late blight of tomato. 

## 4. Discussions

Biosynthesis of nanoparticles using fungi, also called mycosynthesis, is an emerging interdisciplinary science that has the potential to transform conventional farming methods into precision farming [27,28]. Fungi have gained significance in the biosynthesis of nanoparticles due to a number of advantages they possess over other organisms like bacteria and actinomycetes, which are routinely employed for nanoparticle synthesis [29]. Compared to bacteria and actinomycetes, fungal organisms are exceptional producers of proteins, which are secreted into the extracellular medium, thus resulting in the increased yield of nanoparticles [30]. These reductive proteins aid in the extracellular precipitation of nanoparticles and hence allow their direct use in various applications without any requirement of further purification procedures [8]. The secreted proteins also comprise of enzymes that can reduce metal ions in a non-hazardous way [31]. Furthermore, the crystal growth and stability of the nanoparticles can be easily manipulated during the synthesis process due to the slower growth kinetics of fungi [4,31]. Among the various metal based nanoparticles, SeNPs have proven to be an excellent candidate for promising biological applications and are less toxic to the environment [11].

In this study, SeNPs were synthesized by a reduction of 25 mM sodium selenite to elemental selenium, catalyzed by three different fractions of *T. atroviride* broth culture, namely CF, CL, and CW as described by Nandini et al. [19]. The synthesis of nanoparticles was detected by a visual change in color of the extracts from pale yellow to dark brick orange (Figure 1 and Figure 2) after incubating for 24 h at 23 ± 2 °C. Similar synthesis of SeNPs has been demonstrated in earlier studies using *Aspergillus terreus* [32], *Alternaria alternata* [33], *Lentinula edodes* [34], *Fusarium sp*. and *Trichoderma reeii* [35], and *Mariannaea sp*. [36].

The antifungal activities of the SeNPs were evaluated under in vitroconditions using one-month-old chili and tomato leaves inoculated with *Colletotrichum capsici* and *Alternaria solani*. This revealed that the nanoparticles inhibited the mycelial growth of the pathogens at 50 and 100 ppm concentrations (Figure 3 and Figure 4), respectively. The in vitro leaflet assay of silver nanoparticles synthesized from *Ganoderma applanatum* against *Botrytis cinerea* and *Colletotrichum gloeosporioides* on tomato and strawberry leaves was previously investigated [4]. That study also confirmed the inhibitory action against the phytopathogens at a 50 μg/mL concentration. Thus, from these findings, it can be inferred that the SeNPs can be potential tools in the management of plant diseases caused by a wide spectrum of phytopathogens and on different hosts.

In order to study the charge, size, structure, morphology, and chemical compositions of the mycosynthesized SeNPs, characterization studies were carried out by employing techniques such as UV–Vis absorption spectra, dynamic light scattering (DLS), FT-IR, XRD, SEM-EDS, and HR-TEM. The nanoparticles depicted a peak at 260 nm in the UV–Vis absorption spectra (Figure 5), which is analogous with data obtained by Nandini et al. [19]. The Z-average size and zeta potential of the nanoparticles are among the important parameters for the characterization of size and charge, respectively. The Z-average size estimated by DLS provides a reliable measure of average size in a particle size distribution. The magnitude of zeta potential is associated with particle stability, where the higher the zeta potential, the more stable the nanoparticles due to the increased electrostatic repulsions between them. From the present study, it was found that the Z-average size of nanoparticles ranged from 93.2 nm to 98.5 nm and the zeta potential values were in the range of −49.3 mV to −43.7 mV (Figure 6 and Figure 7; Table 2). Hence, it can be clearly interpreted that the nanoparticles synthesized had a uniform size distribution with high stability. However, compared to the results reported by previous studies [19], the nanoparticles synthesized in the present study were more stable due to higher negative values of the zeta potential, and also possessed a more uniform size distribution. These differences may be attributed to the source organism used for the synthesis of SeNPs.

FT-IR is a tool that provides essential details about the chemical interactions involved in the bioreduction of Se^+4^ and the stabilization of SeNPs. The spectrum revealed major absorption bands at 3427.88 cm^−1^ and 1638.83 cm^−1^ (Figure 8), corresponding to the presence of –OH and –NH groups, respectively. The data obtained thus imply that the hydroxyl and the amide groups play an important role in stabilization and the synthetic SeNPs were coated with proteins derived from *T. atroviride*. These data are in line with the results from previous studies that have reported on the functional groups associated with mycosynthesized SeNPs [19,36]. A typical XRD pattern of SeNPs is shown in Figure 9. Seven distinct intense diffraction peaks were observed at 2θ values 23.803°, 29.990°, 31.822°, 41.632°, 43.949°, 45.655°, and 51.994°, corresponding to (1, 0, 0), (1, 1, 0), and (1, 1, 1) sets of lattice planes. These results highlight the crystalline nature of SeNPs in accordance with the XRD data of the SeNPs synthesized from *Mariannaea sp.* [36].

The spherical nature of the SeNPs was verified by SEM (Figure 10). Furthermore, selenium was proven to be the major element present in the nanoparticles as depicted by the energy dispersive spectroscopic graph (Figure 11). Additionally, the HR-TEM micrographs of the nanoparticles exhibited the precise dimensions that ranged from 60.45 nm to 123.19 nm (Figure 12). Recent studies on the synthesis of SeNPs from bacterial [17,37] and fungal sources [19,36] support the data obtained in this study. However, it may also be noted that the size range of the nanoparticles in the present study was much smaller when compared to those in the others. Therefore, based on the data obtained in this study, it can be concluded that nanoparticles of more uniform shape and size were obtained.

One of the most intriguing results from this investigation was the ability of the SeNPs to aggregate *P. infestans*, which is the most devastating pathogen and causes up to 100% yield loss in tomato plants. When the fungal zoospores were treated with different concentrations of SeNPs, an extensive aggregation of zoospores was observed that was visualized using the light microscope (Figure 13 and Figure 14), atomic force microscope (AFM) (Figure 15 and Figure 16) and SEM (Figure 17). It has been reported that the infection of a pathogen on its host requires adhesion of the spores to the host surface [38]. This adhesion permits the pathogen to invade the plant’s surface. Thus, by aggregation of the spores, the infection process of the pathogen can be blocked, thereby limiting the occurrence of disease [38]. Additionally, studies on the cell surface antigens of *Phytophthora* species postulated the presence of chemoreceptors on the cell surface that play an important role in chemotaxis, which further promotes the infection process [39]. There is a probability that by binding to the spores, the nanoparticles have the potential to block the receptors involved in chemotaxis. The genus *Phytophthora* belongs to the class oomycetes, which is phylogenetically different from the true fungi in terms of structure, biology, and pathology. This may have implications for the management of *Phytophthora* infections, as most of the control agents are often not as effective in comparison with that of true fungi [39]. In this context, SeNPs may serve as suitable candidates for the control of *Phytophthora* species. Previously, the aggregation of *Bacillus anthracis* spores by sugar coated single walled carbon nanotubes was investigated by Wang et al. [40], where the nanotubes were chemically modified to target the surface molecules that effectively bind to bacterial spores with divalent cation mediation, bringing about the aggregation of spores that was visualized under electron microscope. The researchers pointed out that such investigations are important in the development of technologies that can detect and decontaminate the disease spreading spores.

## 5. Conclusions

The findings from the present in vitro studies highlight the potential usefulness of mycogenic SeNPs to control a broad spectrum of fungal phytopathogens. The use of such non-toxic biogenic nanoparticles also provides the growing agricultural sector with economic and eco-friendly alternatives to the already existing conventional chemical fertilizers. The aggregation of *P. infestans* zoospores was the most striking finding of the work and this has opened up a new perspective on research into the interactions of oomycete pathogens and nanoparticles. Overall, the present investigation strengthens the use of nanoparticles for the development of newer formulations that can be recommended for integrated disease management in the production of various crop plants, especially tomatoes. 

## Figures and Tables

**Table 1:** Per cent inhibition of *Pyricularia grisea* at different concentrations of mycogenic SeNPs.

Conc. of SeNP (ppm)	Mycelial Diameter (mm)	% of Inhibition
0 (control)	77.2 ± 2.1 ^d^	−
50	64.0 ± 1.4 ^b,c^	17.1 ± 0.4 ^c^
100	52.4 ± 2.0 ^b^	32.12 ± 1.1 ^b^
200	36.8 ± 1.6 ^a^	52.33 ± 0.9 ^a^

Values are the means of three replicates ± SD, means followed by the same letter(s) within the column are not significantly different according to Tukey’s Honestly Significant Difference.

**Table 2:** Z-average size and zeta potential of mycogenic SeNPs as measured by DLS.

SeNPs	Z-Average Size (nm)	Zeta Potential (mV)
CF	98.5 ± 2.9 ^a^	−49.3 ± 1.8 ^a^
CL	93.6 ± 2.1 ^b^	−43.7 ± 0.9 ^b^
CW	93.2 ± 1.7 ^b^	−48.7 ± 2.5 ^a^

Values are the means of three replicates ± SD; means followed by the same letter(s) within the column are not significantly different according to Tukey’s Honestly Significant Difference.
